# The impact of free antiretroviral therapy for pregnant non‐citizens and their infants in Botswana

**DOI:** 10.1002/jia2.26161

**Published:** 2023-10-26

**Authors:** Christina Fennell, Daniel Escudero, Rebecca Zash, Modiegi Diseko, Gloria Mayondi, Judith Mabuta, Tumalano Sekoto, Tendani Gaolathe, Mompati Mmalane, Shahin Lockman, Joseph Makhema, Roger Shapiro

**Affiliations:** ^1^ Department of Epidemiology Harvard T.H. Chan School of Public Health Boston Massachusetts USA; ^2^ Division of Infectious Diseases Beth Israel Deaconess Medical Center Boston Massachusetts USA; ^3^ Botswana Harvard AIDS Institute Partnership Gaborone Botswana; ^4^ Division of Infectious Disease Brigham and Women's Hospital Boston Massachusetts USA; ^5^ Department of Immunology and Infectious Diseases Harvard T.H. Chan School of Public Health Boston Massachusetts USA

**Keywords:** antiretroviral therapy, birth outcomes, botswana, HIV, pregnancy, non‐citizen

## Abstract

**Introduction:**

In December 2019, the Botswana government expanded free antiretroviral therapy (ART) to include non‐citizens. We evaluated the impact of this policy change on antenatal care (ANC), antiretroviral therapy coverage and adverse birth outcomes.

**Methods:**

The Tsepamo Surveillance study collects data at up to 18 delivery sites in Botswana. We compared outcomes in citizens and non‐citizens living with HIV before and after antiretroviral therapy expansion to non‐citizens. Adverse birth outcomes included preterm delivery (PTD) <37 weeks, very preterm delivery (VPTD) <32 weeks, small for gestational age (SGA) <10th percentile, very small for gestational age (VSGA) <3rd percentile, stillbirth and neonatal death. Log‐binomial regression models were constructed to generate risk ratios.

**Results:**

From August 2014 to September 2021, 45,576 (96.5%) citizens and 1513 (3.2%) non‐citizens living with HIV delivered; 954 (62.9%) non‐citizen deliveries were before the antiretroviral therapy expansion, and 562 (37.1%) were after. Non‐citizen ANC attendance among pregnant people living with HIV increased from 79.2% pre‐expansion to 87.2% post‐expansion (*p*<0.001), and became more similar to citizens (96.0% post‐expansion). Non‐citizens receiving any antenatal antiretroviral therapy increased from 65.5% pre‐expansion to 89.9% post‐expansion (*p* < 0.001), also more similar to citizens (97.2% post‐expansion). Infants born to non‐citizens with singleton gestations in the pre‐expansion period had significantly greater risk of PTD (aRR = 1.28, 95% CI, 1.11, 1.46), VPTD (aRR = 1.89, 95% CI, 1.43, 2.44) and neonatal death (aRR = 1.69, 95% CI, 1.03, 2.60), but reduced SGA risk (aRR = 0.75; 95% CI, 0.62, 0.89) compared with citizens. Post‐expansion, greater declines in most adverse outcomes were observed in non‐citizens, with largely similar outcomes between non‐citizens and citizens. Non‐significant differences were observed for non‐citizenship in PTD (aRR = 0.84, 95% CI, 0.66, 1.06), VPTD (aRR = 0.57, 95% CI, 0.28, 1.01), SGA (aRR = 0.91, 95% CI, 0.72, 1.13), VSGA (aRR = 0.87, 95% CI, 0.58, 1.25), stillbirth (aRR = 0.71, 95% CI, 0.35, 1.27) and neonatal death (aRR = 1.35, 95% CI, 0.60, 2.62).

**Conclusions:**

Following the expansion of free antiretroviral therapy to non‐citizens, gaps narrowed in ANC and antiretroviral therapy use in pregnancy between citizens and non‐citizens living with HIV. Disparities in adverse birth outcomes were no longer observed.

## 1 INTRODUCTION

Access to HIV services and treatment may reduce adverse birth outcomes, although the overall benefit may depend on a complex balance between improved maternal health and maintaining a balanced immune response in pregnancy [1, 2, 3, 4, 5]. Approximately 25% of all women between the ages of 15–49 years who are citizens of Botswana are living with HIV, and access to modern antiretroviral treatment (ART) regimens appears to have improved birth outcomes [2, 6, 7]. The Government of Botswana launched a successful free universal antiretroviral therapy programme for all citizens living with HIV in 2002, but until 2019 non‐citizens could only access free HIV care if they obtained citizenship status, married a citizen, had at least one parent who was a citizen, were documented refugees living in a camp or had private healthcare [8, 9, 10]. Approximately 7% of Botswana's population consists of non‐citizens, with many of these individuals migrating from neighbouring countries with high HIV risk, such as Zimbabwe, Zambia and South Africa (with 12.9%, 11.1% and 19.1% HIV prevalence, respectively) [6, 9, 11, 12, 13, 14].

According to a study by Marukutira et al., only 29% of migrants in Botswana had personal health insurance or could afford to pay for HIV care, only 1% had received HIV treatment through a refugee camp and migrants had worse health outcomes than citizens [9]. In addition to treatment costs, negative encounters with healthcare professionals and fear of security agents were also noted as barriers to receiving care [15]. Non‐citizens have historically been less likely to receive antenatal care (ANC) than Botswana citizens, more likely to deliver at home and receive care later in pregnancy due to healthcare barriers, and more likely than citizens to experience adverse birth outcomes [16]. These findings are consistent with similar studies conducted in Europe [17, 18, 19, 20].

Beginning in December 2019, all non‐citizens were allowed access to free antiretroviral therapy in Botswana [21]. To implement this change, the Ministry of Health and Wellness disseminated a government directive that authorized the distribution of treatment to non‐citizens [22]. Using data from the Tsepamo study, a large birth outcomes surveillance programme in Botswana, we evaluated the impact of the free antiretroviral therapy‐policy expansion on ANC attendance, antiretroviral therapy coverage and adverse birth outcomes among infants born to non‐citizens.

## 2 METHODS

### 2.1 Study population

This secondary analysis of data from the Tsepamo study included birth outcomes that occurred from up to 18 maternity sites at government facilities throughout Botswana. Before July 2018, data collection occurred at eight government maternity wards and accounted for approximately 45% of all births in Botswana [2]. The study sites later expanded to 18 maternity wards, accounting for approximately 72% of all births in the country [23]. Data on deliveries included all in‐hospital live and stillbirths ≥24 weeks gestation. In Botswana, approximately 95% of all deliveries occur in a hospital or medical facility [24]. As the Tsepamo study began in August 2014, the pre‐antiretroviral therapy policy expansion period was defined as August 2014–November 2019 and the post‐antiretroviral therapy policy expansion period was defined as December 2019–September 2021. Because multiple births may be associated with adverse birth outcomes, only singleton gestations were included for the analyses of adverse birth outcomes [25].

### 2.2 Data extraction

Extracted data from maternal obstetric records included demographic information, maternal medical history, ANC information, inpatient care during labour and delivery, medical diagnoses made in pregnancy, adverse birth outcomes, maternal HIV status and HIV treatment information. Citizenship status is determined with the use of national identity cards (Omang) for citizens, and non‐citizens present their country of origin documentation when accessing healthcare services. Infant information included gestational age, birth weight, sex and admission to the neonatal unit. All diagnoses included in the analyses were recorded as documented by the treating physician or midwife. The date of the last menstrual period was used to estimate gestational age. If the date of the last menstrual period was unknown, healthcare providers used second‐ or third‐trimester ultrasound or fundal height measurement to determine the gestational age. Maternal obstetric records for all births were reviewed at the time of discharge and clinical data from the obstetric cards were transcribed into a REDCap database [2, 23].

### 2.3 Adverse birth outcomes

All adverse birth outcomes were determined by delivery nurses at the maternity wards and the data were abstracted from their antenatal records [2, 23]. Adverse birth outcomes included preterm delivery (PTD) (<37 weeks gestational age), very preterm delivery (VPTD) (<32 weeks gestational age), stillbirth (combined Apgar score of 0 and includes macerated and fresh stillbirths) and neonatal death (in‐hospital infant death <28 days). Small for gestational age (SGA) (<10th percentile) and very SGA (<3rd percentile) were defined based on the intergrowth‐21 norms [26, 27].

### 2.4 Statistical analyses

Proportions of maternal demographics, ANC attendance, HIV status, antiretroviral therapy uptake and adverse birth outcomes were determined and compared between citizens and non‐citizens living with HIV before and after the December 2019 antiretroviral therapy expansion to non‐citizens. Based on subject‐matter knowledge, identified covariates for our adjusted analyses included maternal age, education, occupation, parity and marital status. As only citizens were permitted access to free antiretroviral therapy before December 2019, pregnant citizens living with HIV were selected as the primary referent group to demonstrate the impact of reduced barriers to antiretroviral therapy on the health of pregnant non‐citizens living with HIV and their infants. Sensitivity analyses were conducted to explore potential associations between citizenship status and adverse birth outcomes among pregnant people without HIV, to account for the impact of COVID‐19 on adverse birth outcomes, to account for multiple pregnancies and to evaluate any potential impact due to missing data. Log‐binomial regression models for the primary and sensitivity analyses were used to obtain crude and adjusted risk ratio (aRR) estimates with 95% confidence intervals. *p*‐Values were computed using two‐sided tests at the α  =  0.05 significance level. All statistical analyses were performed using RStudio version 1.4.1717 software.

### 2.5 Ethical approval

Intuitional approval for this study was provided by the Health Research and Development Committee in Botswana and the institutional review board of Harvard T.H. Chan School of Public Health in Boston, Massachusetts. As data were collected anonymously and via chart abstraction, maternal consent was waived.

## 3 RESULTS

**Figure 1:**
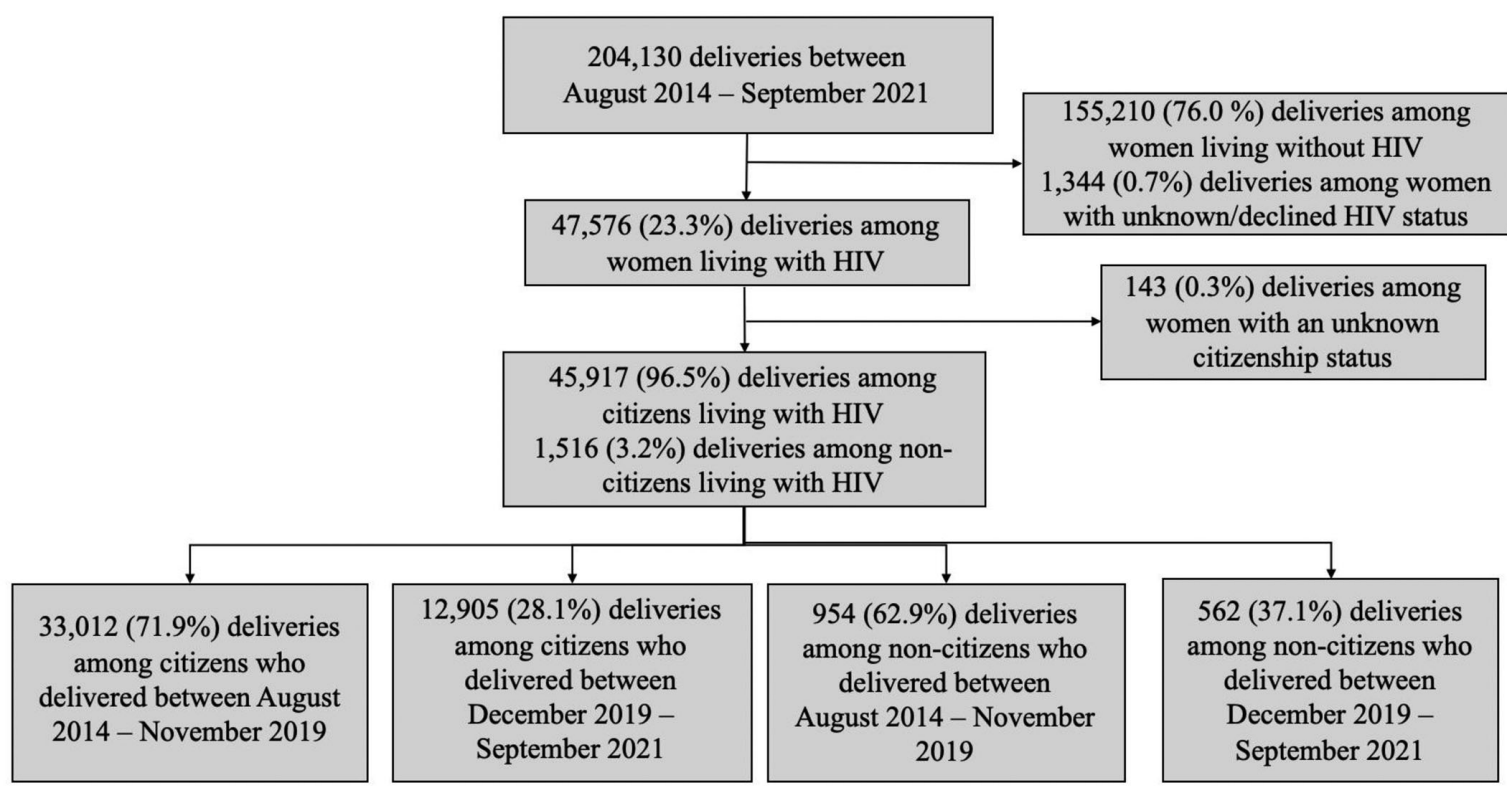
Flow chart of study population.

From August 2014 to September 2021, there were 204,130 live deliveries and stillbirths recorded in the Tsepamo birth surveillance study (Figure 1), including 195,839 (95.9%) to citizens and 7607 (3.7%) to non‐citizens (Table 1). In total, 47,576 (23.3%) deliveries were among pregnant people known to be living with HIV, including 47,443 (23.2%) with known citizenship status. Among the total number of pregnant people living with HIV with a known citizenship status, 45,917 (96.8%) deliveries occurred among citizens, and 1516 (3.2%) deliveries occurred among non‐citizens.

**Table 1:** Maternal characteristics of all citizens versus non‐citizens living with or without HIV in the pre‐antiretroviral therapy expansion period (August 2014–November 2019 delivery dates) and the post‐antiretroviral therapy expansion period (December 2019–September 2021 delivery dates)

	Citizens			Non‐citizens		
	Pre‐ART expansion (*n* = 137,814)	Post‐ART expansion (*n* = 58,025)	*p*‐value	Pre‐ART expansion (*n* = 4711)	Post‐ART expansion (*n* = 2896)	*p*‐value
Maternal age, years (median, IQR)	26 (22, 32)	27 (22, 33)	<0.001	29 (24, 34)	28 (23, 34)	<0.001
Education^a^, ^b^			<0.001			0.39
Primary/none	9844 (7.1)	3808 (6.6)		525 (11.1)	350 (12.1)	
Secondary/tertiary educ.	125,220 (90.9)	53,118 (91.5)		3698 (78.5)	2314 (79.9)	
Occupation			<0.001			0.04
Non‐salaried/housewife	76,809 (55.7)	33,450 (57.6)		3105 (65.9)	1968 (68.0)	
Student	9161 (6.6)	3196 (5.5)		65 (1.4)	22 (0.8)	
Salaried	46,441 (33.7)	19,326 (33.3)		1037 (22.0)	651 (22.5)	
Marital status			<0.001			<0.01
Single	120,966 (87.8)	51,160 (88.2)		2038 (43.3)	1362 (47.0)	
Married	12,331 (8.9)	4761 (8.2)		2368 (50.3)	1344 (46.4)	
Divorced/widowed	428 (0.3)	160 (0.3)		16 (0.3)	10 (0.3)	
Parity			<0.001			0.02
Primiparous	53,726 (39.0)	21,353 (36.8)		1086 (23.1)	751 (25.9)	
Multiparous 2–4	71,285 (51.7)	30,499 (52.6)		3212 (68.2)	1907 (65.8)	
Grand multiparous ≥5	12,400 (9.0)	6006 (10.4)		369 (7.8)	213 (7.4)	
Maternity site			<0.001			<0.001
Rural	79,715 (57.8)	40,268 (69.4)		1462 (31.0)	1440 (49.7)	
Urban	58,098 (42.2)	17,756 (30.6)		3249 (69.0)	1456 (50.3)	
Median ANC visits (IQR)	10 (7, 12)	10 (7, 12)	<0.001	7 (2, 10)	7 (3,10)	<0.001
One or more ANC visit			0.91			<0.001
No	3295 (2.4)	1384 (2.4)		798 (16.9)	326 (11.3)	
Yes	133,358 (96.8)	56,212 (96.9)		3842 (81.6)	2533 (87.5)	
Singleton births			0.44			0.53
No	2068 (1.5)	844 (1.5)		105 (2.2)	71 (2.5)	
Yes	135,745 (98.5)	57,181 (98.5)		4606 (97.8)	2825 (97.5)	
Maternal HIV status			<0.001			0.047
Negative	104,110 (75.5)	44,900 (77.4)		3463 (73.5)	2296 (79.3)	
Positive	33,012 (24.0)	12,905 (22.2)		954 (20.3)	562 (19.4)	
Unknown	692 (0.5)	220 (0.4)		294 (6.2)	38 (1.3)	

Abbreviations: ANC, antenatal care; ART, antiretroviral therapy; IQR, interquartile range.

^a^ Percentages may not add to 100% due to rounding.

^b^ Missing data in the pre‐antiretroviral therapy expansion period for citizens included: 55 (0.04) for maternal age, 2750 (2.0%) for education, 5403 (3.9%) for occupation, 4059 (2.9%) for marital status, 403 (0.3%) for parity, 1 (0.0%) for maternity site, 1161 (0.8%) for one or more clinic visits and 1 (0.0%) for singleton births. Missing data in the post‐antiretroviral therapy expansion period for citizens included: 36 for maternal age (0.06), 1099 (1.9%) for education, 2053 (3.5%) for occupation, 1944 (3.4%) for marital status, 167 (0.3%) for parity, 1 (0.0%) for maternity site and 429 (0.7%) for one or more clinic visits. Missing data in the pre‐antiretroviral therapy expansion period for non‐citizens included: 4 (0.08) for maternal age, 488 (10.4%) for education, 504 (10.7%) for occupation, 289 (6.1%) for marital status, 44 (0.9%) for parity and 71 (1.5%) for one or more clinic visits. Missing data in the post‐antiretroviral therapy expansion period for non‐citizens included: 2 (0.07) for maternal age, 232 (8.0%) for education, 255 (8.8%) for occupation, 180 (6.2%) for marital status, 25 (0.9%) for parity and 37 (1.3%) for one or more clinic visits.

### 3.1 Baseline characteristics pre‐ versus post‐antiretroviral therapy expansion

Among citizens, 137,814 (70.4%) deliveries occurred in the pre‐antiretroviral therapy expansion period and 58,025 deliveries (29.6%) in the post‐antiretroviral therapy expansion period (Table 1). In the pre‐antiretroviral therapy expansion period, the median maternal age for pregnant citizens was 26 years (IQR: 22, 32) compared with 27 years (IQR: 22, 33) in the post‐antiretroviral therapy expansion period. Other maternal demographics remained largely similar between the pre‐ and post‐antiretroviral therapy expansion periods. Exceptions included that citizens in the post‐antiretroviral therapy expansion period became more likely to deliver at a rural maternity site (57.8% vs. 69.4%) and there was a modest decline in the proportion of pregnant people living with HIV (24.0% vs. 22.2%) (all *p*‐values < 0.05).

There were 7607 deliveries among non‐citizens during the entire study period, with 4711 (61.9%) deliveries in the pre‐antiretroviral therapy expansion period and 2896 deliveries (38.1%) in the post‐antiretroviral therapy expansion period. In the pre‐antiretroviral therapy expansion period, the median age for pregnant non‐citizens was 29 years (IQR: 24, 34) compared with 28 years (IQR: 23, 34) in the post‐antiretroviral therapy expansion period. Compared with the pre‐antiretroviral therapy expansion period, non‐citizens in the post‐antiretroviral therapy expansion period were less likely to be married (50.3% vs. 46.4%), but more likely to be primiparous (23.1% vs. 25.9%), to deliver at a rural maternity site (31.0% vs. 49.7%), and to have at least one ANC visit (81.6% vs. 87.5%) (all *p*‐values < 0.05).

### 3.2 HIV outcomes and antiretroviral therapy uptake pre‐ versus post‐antiretroviral therapy expansion

**Figure 2:**
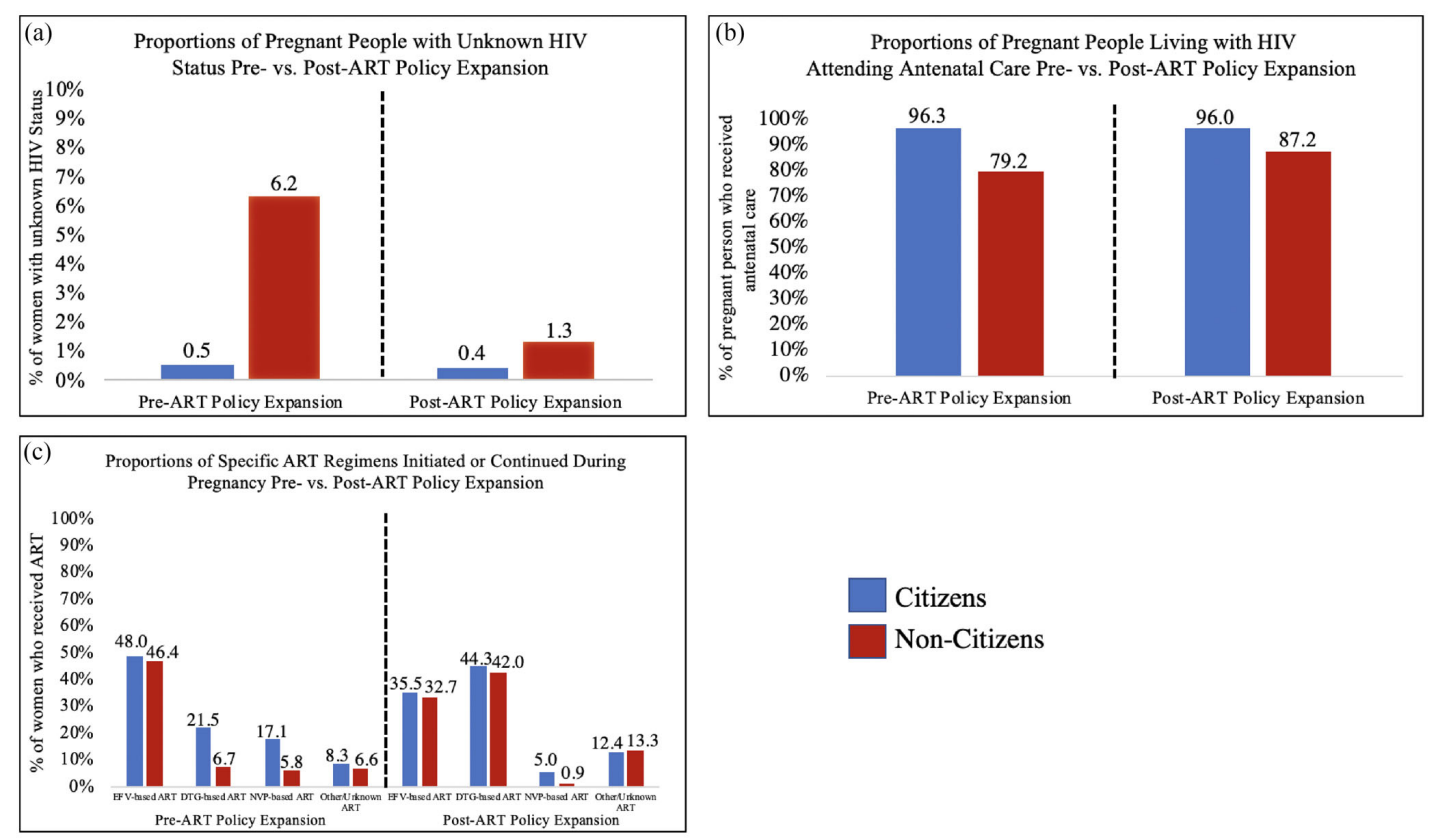
Proportions of antenatal care and maternal HIV treatment outcomes among citizens and non‐citizens before and after the antiretroviral therapy expansion to non‐citizens in December 2019.

Several characteristics related to HIV and antiretroviral therapy differed between the pre‐ and post‐antiretroviral therapy expansion eras, and are highlighted below. The proportion of non‐citizens with unknown HIV status decreased in post‐antiretroviral therapy policy expansion period (6.2% vs. 1.3%) (*p* < 0.001), whereas the proportion of citizens with unknown HIV status generally remained unchanged (0.5% vs. 0.4%) (*p* = 0.02) (Figure 2a). For non‐citizens living with HIV, the proportion of pregnant people who received any ANC (at least one antenatal visit) was 79.2% in the pre‐antiretroviral therapy policy expansion period and increased to 87.2% in the post‐antiretroviral therapy policy expansion period (*p* < 0.001) (Figure 2b). In comparison, the proportion of citizens living with HIV who received any ANC remained generally constant in the pre‐ and post‐antiretroviral therapy policy expansion periods at approximately 96.0%. In the pre‐antiretroviral therapy expansion period, 65.5% of non‐citizens received any antiretroviral therapy, with only 6.7% receiving a dolutegravir (DTG)‐based regimen (Figure 2c). After the policy expansion in December 2019, the proportion of non‐citizens receiving antiretroviral therapy increased markedly, to 89.9% (*p* < 0.001), which narrowed the gap with citizens (97.2%). After December 2019, the proportion of non‐citizens and citizens receiving DTG‐based antiretroviral therapy was similar (42.0% vs. 44.3%).

### 3.3 Adverse birth outcomes pre‐ versus post‐antiretroviral therapy expansion among infants born to pregnant people with HIV

**Figure 3:**
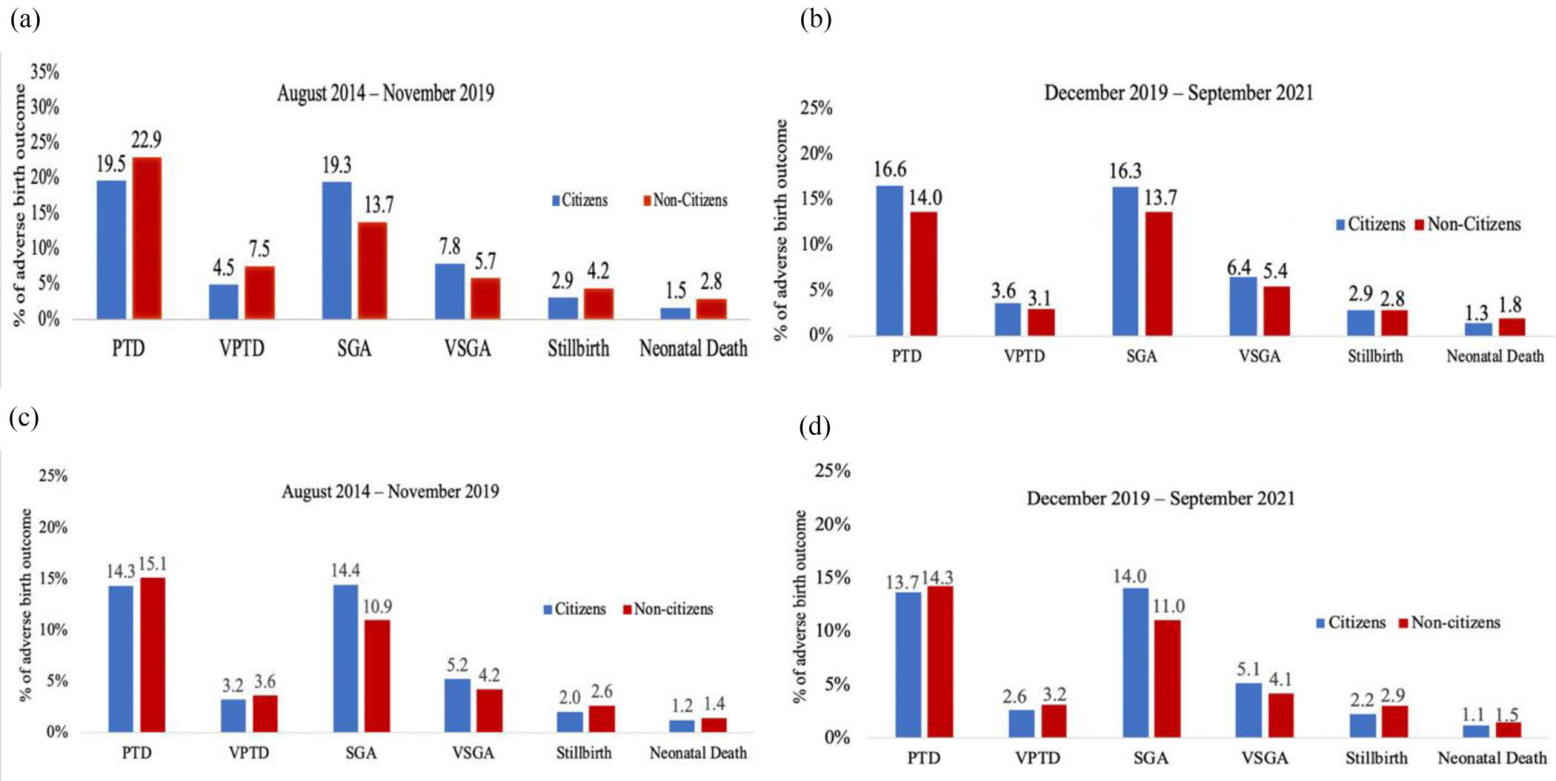
Proportions of adverse birth outcomes among citizens versus non‐citizens with singleton infants in the pre‐antiretroviral therapy expansion period (August 2014–November 2019 delivery dates) and the post‐antiretroviral therapy expansion period (December 2019–September 2021 delivery dates). Panels a and b display the proportion of adverse birth outcomes among pregnant people living with HIV, and panels c and d display the proportion of adverse birth outcomes among pregnant people without HIV.Abbreviations: PTD, preterm delivery (<37 weeks gestational age); SGA, small for gestational age (<10th percentile for gestational age); VPTD, very preterm delivery (<32 weeks gestational age); VSGA, very small for gestational age (<3rd percentile for gestational age).

In the pre‐antiretroviral therapy expansion era, the proportions of PTD, VPTD, stillbirth and neonatal death were higher among singleton infants born to non‐citizens with HIV (*n* = 926) than to citizens with HIV (*n* = 32,394) (Figure 3a). SGA and VSGA were lower among singletons born to non‐citizens with HIV during this period. After adjusting for potential confounders, maternal age, education, occupation, marital status and parity, the relative risks for these associations between non‐citizenship and adverse pregnancy outcome were significant for PTD (aRR = 1.28, 95% CI, 1.11, 1.46), VPTD (aRR = 1.89, 95% CI, 1.43, 2.44) and neonatal death (aRR = 1.69, 95% CI, 1.03, 2.60), and there was a potential difference observed for stillbirths (aRR = 1.38, 95% CI, 0.95, 1.94) (Table 2). There was a significant protective association between non‐citizenship and SGA (aRR = 0.75; 95% CI, 0.62, 0.89), but this was not significant for VSGA (aRR = 0.81; 95% CI, 0.61, 1.06).

**Table 2:** Adverse birth outcomes in Botswana among singleton infants born to citizens and non‐citizens living with HIV, before and after the antiretroviral therapy expansion to non‐citizens in December 2019

	August 2014–November 2019				December 2019–September 2021			
	Citizen *n* = 32,394 (%)	Non‐citizen *n* = 926 (%)	RR, 95% CI	aRR^a^, 95% CI	Citizen *n* = 12,666 (%)	Non‐citizen *n* = 541 (%)	RR, 95% CI	aRR^a^, 95% CI
PTD	6312 (19.5)	212 (22.9)	1.22 (1.08, 1.37)	1.28 (1.11, 1.46)	2107 (16.6)	76 (14.0)	0.87 (0.70, 1.07)	0.84 (0.66, 1.06)
VPTD	1462 (4.5)	69 (7.5)	1.72 (1.35, 2.15)	1.89 (1.43, 2.44)	462 (3.6)	17 (3.1)	0.89 (0.53, 1.38)	0.57 (0.28, 1.01)
SGA	6264 (19.3)	127 (13.7)	0.74 (0.63, 0.87)	0.75 (0.62, 0.89)	2068 (16.3)	74 (13.7)	0.86 (0.69, 1.06)	0.91 (0.72, 1.13)
VSGA	2513 (7.8)	53 (5.7)	0.77 (0.58, 0.99)	0.81 (0.61, 1.06)	811 (6.4)	29 (5.4)	0.86 (0.59, 1.21)	0.87 (0.58, 1.25)
Stillbirth	935 (2.9)	39 (4.2)	1.46 (1.05, 1.97)	1.38 (0.95, 1.94)	370 (2.9)	15 (2.8)	0.95 (0.54, 1.52)	0.71 (0.35, 1.27)
Neonatal death	500 (1.5)	26 (2.8)	1.85 (1.22, 2.67)	1.69 (1.03, 2.60)	164 (1.3)	10 (1.8)	1.43 (0.71, 2.40)	1.35 (0.60, 2.62)

Abbreviations: aRR, adjusted risk ratio; PTD, preterm delivery (<37 weeks gestational age); RR, risk ratio; SGA, small for gestational age (<10th percentile for gestational age); VPTD, very preterm delivery (<32 weeks gestational age); VSGA, very small for gestational age (<3rd percentile for gestational age).

^a^ Adjusted for maternal age, education, occupation, marital status and parity.

In the post‐antiretroviral therapy policy expansion period, none of the adverse birth outcomes were significantly higher among singleton infants born to non‐citizens (*n* = 541) compared with citizens (*n* = 12,666), and there were greater overall declines in adverse birth outcomes among singletons born to non‐citizens (Figure 3b). The adjusted model results showed that the adverse birth outcomes did not differ in the post‐antiretroviral therapy era for non‐citizens compared with citizens: PTD (aRR = 0.83, 95% CI, 0.66, 1.06), VPTD (aRR = 0.57, 95% CI, 0.28, 1.01), SGA (aRR = 0.91, 95% CI, 0.72, 1.13), VSGA (aRR = 0.87, 95% CI, 0.58, 1.25) stillbirth (aRR = 0.70, 95% CI, 0.35, 1.27) and neonatal death (aRR = 1.35, 95% CI, 0.60, 2.62).

### 3.4 Adverse birth outcomes pre‐ versus post‐antiretroviral therapy expansion among infants born to pregnant people without HIV

As an external control, to account for possible changes in demographics among non‐citizens living with HIV, we conducted a similar analysis using the same study eras among non‐citizens living without HIV (Figure S1). We found that the proportion of HIV‐negative non‐citizens with adverse birth outcomes did not significantly differ pre versus post‐antiretroviral therapy policy expansion for any category.

### 3.5 Sensitivity analyses

Our first sensitivity analysis consisted of generating log‐binomial regression models to explore potential associations between non‐citizenship status and adverse birth outcomes among pregnant people without HIV (Table S1). The only harmful association identified was for PTD in the pre‐free antiretroviral therapy expansion period, which became non‐significant in the post‐free antiretroviral therapy expansion period. To account for potential bias due to COVID‐19, we generated our original models and excluded data from 2021, which led to findings consistent with our main results (Table S2). We then restricted our data to first‐time pregnancies to avoid potential bias from multiple pregnancies during our study period (Table S3). While our sample size was drastically reduced, the general findings remained consistent in direction and magnitude. To evaluate potential bias from missing data among non‐citizens, we assigned missing values to no/none, where applicable, and our results remained consistent with our main results (Tables S4 and S5).

## 4 DISCUSSION

We evaluated the impact of expanding free antiretroviral therapy coverage to non‐citizens and found that following this policy shift in Botswana, gaps narrowed between citizens and non‐citizens in ANC attendance, antiretroviral therapy uptake and adverse birth outcomes. While causality cannot be proven in this observational study, these temporal associations suggest that greater access to modern antiretroviral therapy regimens used in pregnancy may have reduced adverse birth outcomes and should be considered to be a part of a larger strategy for promoting infant health.

We are unaware of similar studies that explore the impact of expanding free antiretroviral therapy to include non‐citizens on adverse birth outcomes. However, among studies related to the expansion of antiretroviral therapy for non‐citizens living with HIV, there is evidence to support that although non‐citizens tend to present to care with more advanced HIV, when non‐citizens living with HIV are linked to care, their level of retention in care and viral suppression are similar to citizens living with HIV who are linked to care [28, 29, 30, 31, 32]. Importantly, our study found that the proportion with unknown HIV status among pregnant non‐citizens decreased substantially in the post‐antiretroviral therapy policy expansion period. In addition, a higher proportion of pregnant non‐citizens were linked to antiretroviral therapy, and accessed ANC services. These findings align with a study using data from 10 European countries reporting that a larger proportion of non‐citizens were diagnosed with HIV late in pregnancy compared to citizens, suggesting that having greater access to medical care during pregnancy can lead to faster knowledge regarding HIV status, quicker linkage to HIV treatment and potentially improved birth outcomes [33].

By evaluating the impact of the Government of Botswana's policy change, we also gained insight into the role of antiretroviral therapy for improving birth outcomes in pregnant people with HIV in the modern era. Studies that have demonstrated the consequences of receiving antenatal antiretroviral therapy compared to not receiving any antiretroviral therapy during pregnancy have been limited. For ethical reasons, comparing no antiretroviral therapy with any antiretroviral therapy in the context of a randomized trial is infeasible, and observational studies that attempt to emulate this target trial are vulnerable to immortal time bias [34, 35, 36]. The PROMISE trial explored the impact of receiving combined zidovudine‐based antiretroviral therapy or tenofovir‐based antiretroviral therapy during pregnancy compared with zidovudine alone [37]. It was found that receiving combined antiretroviral therapy during pregnancy was associated with an increase in adverse birth outcomes compared to the control arm. These results aligned with the first trial that explored the use of zidovudine‐alone during pregnancy [38]. However, these trials used legacy regimens that are known to be associated with adverse birth outcomes [2, 39, 40, 41, 42]. More recent studies have shown that the use of updated regimens during pregnancy, such as efavirenz (EFV)‐containing regimens and particularly DTG‐containing antiretroviral therapy, is associated with a lower risk of adverse birth outcomes compared to legacy regimens [2, 41, 42]. In our analysis of the specific regimens used among citizens and non‐citizens during the study period (Figure 2c), notable findings included the decreased use of nevirapine (NVP)‐based antiretroviral therapy (a known legacy regimen associated with a greater risk of adverse birth outcomes) among both citizens and non‐citizens, and greater use of any antiretroviral therapy (and specifically DTG‐based antiretroviral therapy) among non‐citizens after the policy expansion. These findings lend support to previous studies that encouraged the use of more modern antiretroviral therapy regimens during pregnancy.

It is possible that the reported improvements in adverse birth outcomes among non‐citizens are not solely due to increased access to antiretroviral therapy or modern antiretroviral therapy regimens. Other factors, particularly increased linkage to ANC among non‐citizens, are likely to have a beneficial impact on adverse birth outcomes as well [43, 44, 45]. Yet, there is support for the beneficial impact of increased access to free antiretroviral therapy on pregnancy outcomes, as we did not see the same level of improvement in adverse birth outcomes among pregnant non‐citizens without HIV (Figures 3c and d). Moreover, our results demonstrated that ANC utilization among pregnant non‐citizens without HIV did not meaningfully change in the post‐free antiretroviral therapy expansion period, and the gap between people living with HIV or without HIV among pregnant non‐citizens narrowed in the post‐free antiretroviral therapy expansion period (Table S6). Therefore, it is likely that the greater use of ANC services among non‐citizens living with HIV is related to the expanded access to free antiretroviral therapy. As such, it is important to highlight the need for greater efforts to link non‐citizens living with HIV to both ANC and antiretroviral therapy, as there is well‐documented evidence that access to antenatal services is associated with improved birth outcomes [43, 44, 45].

Another notable finding was that citizens experienced higher proportions of SGA compared with non‐citizens, and there was an apparent protective association between non‐citizenship status and SGA in the pre‐antiretroviral therapy expansion period among pregnant people living with HIV. A previous Tsepamo study reported that pregnant people on efavirenz or DTG‐based antiretroviral therapy combined with tenofovir tend to have lower weight gain compared to pregnant people without HIV, thus differences in antiretroviral therapy regimens between citizens and non‐citizens might explain our findings (low maternal weight gain is associated with SGA) [46, 47]. However, a protective association also occurred among those without HIV for SGA and VSGA (Table s1). Possible explanations for this larger group include that infants born in Botswana are reportedly smaller compared with infants born in other countries [48]. We also cannot exclude the possibility that the non‐citizens who delivered at government facilities differed from citizens in other health parameters that made them less likely to be SGA compared with citizens. Lastly, this finding could be a consequence of having a limited sample of non‐citizens who delivered in Botswana.

The strengths of our study included a large sample size of pregnant people, well‐defined adverse birth outcomes, and detailed ANC and antiretroviral therapy information. Additionally, our sensitivity analyses support our findings (Tables S1 and S2). Moreover, to our knowledge, this is the first study in Botswana to explore the impact of free antiretroviral therapy among pregnant non‐citizens and possibly the first study to explore this research question. Limitations include that the associations between improved outcomes and the antiretroviral therapy expansion in Botswana do not prove causality as there are other possible reasons for improved outcomes over time among non‐citizens living with HIV, although such improvements were not observed in citizens or in non‐citizens without HIV. For example, while we identified an increase in engagement in the healthcare system by non‐citizens, it is important to note that well‐documented barriers to care may exist among non‐citizens (including negative encounters with healthcare professionals or fear of authorities) [9, 10, 15, 49]. As a result, we do not have the documentation status of non‐citizens, and we acknowledge that our sample of non‐citizens may have largely included those with fewer structural barriers to receiving care at government facilities, possibly leading to better infant health outcomes (biasing results towards the null). Moreover, due to our belief that antiretroviral therapy regimens may be a time‐varying confounder (as non‐citizens are less likely to receive any antiretroviral therapy/receive an updated antiretroviral therapy regimen, which may impact their pregnancy/maternal health status as a non‐citizen vs. citizen, and citizenship status in addition to being on any antiretroviral therapy/specific antiretroviral therapy regimens are associated with adverse birth outcomes), the use of any antiretroviral therapy/specific antiretroviral therapy regimens was not included in our adjusted analyses. There were minor differences in the missingness of some demographic covariates between citizens and non‐citizens, but our sensitivity analyses did not result in notable changes in our findings. Lastly, the impact of the policy expansion on HIV transmission to infants cannot be determined using these data alone, and our data set did not include deliveries or miscarriages <24 weeks gestation.

## 5 CONCLUSIONS

In conclusion, following the expansion of free antiretroviral therapy to non‐citizens in Botswana, there were temporal improvements in HIV diagnoses, antiretroviral therapy uptake in pregnancy with a DTG‐based regimen and adverse birth outcomes. These improvements were noted in non‐citizens living with HIV but not among other exposure groups, suggesting that antiretroviral therapy may play a role in narrowing previous gaps in health outcomes. While Botswana is fortunate to have a mature antiretroviral therapy programme with very high coverage, these findings may also be applicable in other regions where barriers exist to improving antiretroviral therapy uptake in pregnancy, adding urgency to efforts towards universal access to modern antiretroviral therapy regimens.

## COMPETING INTERESTS

There are no reported competing interests.

## AUTHORS’ CONTRIBUTIONS

CF, DE and RS designed the study and performed the analysis. RS, MD, GM, JM, TS, TG, MM, SL and JM helped to oversee and operate the Tsepamo study and provided data for this study. CF, DE and RS drafted the manuscript; RZ, MD, SL, JM and TS reviewed and contributed to the development of the manuscript, and all authors read and approved the final manuscript.

## FUNDING

The study was supported by the National Institute of Child Health and Human Development (NICHD/NIH) (R01HD080471 and R01HD095766).

## Supporting information

Supporting Information

Supporting Information

## Data Availability

The data set analysed for this research is not available.
