# Supplementary material for: The impact of free antiretroviral therapy for pregnant non‐citizens and their infants in Botswana
Source: J Int AIDS Soc. 2023 Oct 26;26(10):e26161. doi: 10.1002/jia2.26161 (PMC10603275; doi:10.1002/jia2.26161)
Supplement: Supplementary file 1 — Supporting Information [file JIA2-26-e26161-s002.docx]

**Figure S1. Proportions of Adverse Birth Outcomes Among Non-Citizens Without HIV Pre- vs. Post-ART Policy Expansion. Figure Legend:** Abbreviations: PTD, preterm delivery (<37 weeks gestational age); VPTD, very preterm delivery (<32 weeks gestational age); SGA, small for gestational age (<10^th^ percentile for gestational age); VSGA, very small for gestational age (<3^rd^ percentile for gestational age). This figure displays the changes in the adverse birth outcomes among non-citizens after the ART policy expansion.
