# Supplementary material for: The impact of free antiretroviral therapy for pregnant non‐citizens and their infants in Botswana
Source: J Int AIDS Soc. 2023 Oct 26;26(10):e26161. doi: 10.1002/jia2.26161 (PMC10603275; doi:10.1002/jia2.26161)
Supplement: Supplementary file 2 — Supporting Information [file JIA2-26-e26161-s001.docx]

**Table S1. Adverse birth outcomes in Botswana among citizens and non-citizens, before and after ART expansion in December 2019 among pregnant people without HIV [singleton births]**

|  | August 2014 - November 2019 | | | | December 2019 – September 2021 | | | |
| --- | --- | --- | --- | --- | --- | --- | --- | --- |
|  | Citizen  n =102,669 (%) | Non-Citizen  n = 3,393 (%) | RR,  95% CI | aRR^†^,  95% CI | Citizen  n = 44,297 (%) | Non-Citizen  n= 2,248 (%) | RR,  95% CI | aRR^†^,  95% CI |
| PTD^‡^ | 14,701 (14.3) | 514 (15.1) | 1.08 (0.99, 1.16) | 1.12 (1.02, 1.23) | 6,068 (13.7) | 322 (14.3) | 1.08 (0.97, 1.19) | 1.09 (0.97, 1.22) |
| VPTD | 3,298 (3.2) | 121 (3.6) | 1.13 (0.94, 1.34) | 1.08 (0.86, 1.33) | 1,160 (2.6) | 71 (3.2) | 1.24 (0.97, 1.56) | 1.11 (0.82, 1.47) |
| SGA | 14,777 (14.4) | 370 (10.9) | 0.77 (0.70, 0.85) | 0.82 (0.73, 0.91) | 6,205 (14.0) | 247 (11.0) | 0.81 (0.72, 0.91) | 0.89 (0.77, 1.01) |
| VSGA | 5,367 (5.2) | 143 (4.2) | 0.82 (0.69, 0.96) | 0.82 (0.67, 0.98) | 2,268 (5.1) | 92 (4.1) | 0.83 (0.67, 1.01) | 0.87 (0.69, 1.08) |
| Stillbirth | 2,087 (2.0) | 88 (2.6) | 1.28 (1.03, 1.56) | 1.20 (0.94, 1.52) | 978 (2.2) | 66 (2.9) | 1.33 (1.03, 1.68) | 1.23 (0.91, 1.62) |
| Neonatal Death | 1,273 (1.2) | 49 (1.4) | 1.17 (0.87, 1.54) | 1.12 (0.78, 1.55) | 476 (1.1) | 33 (1.5) | 1.38 (0.95, 1.92) | 1.15 (0.71, 1.75) |

†Adjusted for maternal age, education, occupation, marital status, and parity

‡ Abbreviations: PTD, preterm delivery (<37 weeks gestational age); VPTD, very preterm delivery (<32 weeks gestational age); SGA, small for gestational age (<10^th^ percentile for gestational age); VSGA, very small for gestational age (<3^rd^ percentile for gestational age).

|  | August 2014 - November 2019 | | | | December 2019 – December 2020 | | | |  |
| --- | --- | --- | --- | --- | --- | --- | --- | --- | --- |
|  | Citizen  n =102,669 (%) | Non-Citizen  n = 3,393 (%) | RR,  95% CI | aRR^†^,  95% CI | Citizen  n = 8,491 (%) | Non-Citizen  n = 321 (%) | RR,  95% CI | aRR^†^,  95% CI | |
| PTD^‡^ | 14,701 (14.3) | 514 (15.1) | 1.08 (0.99, 1.16) | 1.12 (1.02, 1.23) | 1,427 (16.8) | 45 (14.0) | 0.87 (0.65, 1.12) | 0.81 (0.58, 1.09) | |
| VPTD | 3,298 (3.2) | 121 (3.6) | 1.13 (0.94, 1.34) | 1.08 (0.86, 1.33) | 315 (3.7) | 10 (3.1) | 0.87 (0.44, 1.53) | 0.67 (0.29, 1.32) | |
| SGA | 14,777 (14.4) | 370 (10.9) | 0.77 (0.70, 0.85) | 0.82 (0.73, 0.91) | 1,348 (15.9) | 40 (12.5) | 0.81 (0.60, 1.07) | 0.94 (0.69, 1.25) | |
| VSGA^§^ | 5,367 (5.2) | 143 (4.2) | 0.82 (0.69, 0.96) | 0.82 (0.67, 0.98) | 515 (6.1) | 18 (5.6) | 0.96 (0.58, 1.46) | 1.02 (0.59, 1.66) | |
| Stillbirth | 2,087 (2.0) | 88 (2.6) | 1.28 (1.03, 1.56) | 1.20 (0.94, 1.52) | 221 (2.6) | 6 (1.9) | 0.72 (0.28, 1.46) | 0.52 (0.16, 1.23) | |
| Neonatal Death^§^ | 1,273 (1.2) | 49 (1.4) | 1.17 (0.87, 1.54) | 1.12 (0.78, 1.55) | 105 (1.2) | 8 (2.5) | 2.00 (0.90, 3.81) | 1.94 (0.74, 4.26) | |

**Table S2. Adverse birth outcomes in Botswana among citizens and non-citizens, before and after ART expansion in December 2019 among pregnant people living with HIV [excluding 2021 data]**

†Adjusted for maternal age, education, occupation, marital status, and parity

‡ Abbreviations: PTD, preterm delivery (<37 weeks gestational age); VPTD, very preterm delivery (<32 weeks gestational age); SGA, small for gestational age (<10^th^ percentile for gestational age); VSGA, very small for gestational age (<3^rd^ percentile for gestational age).

Adjusted risk ratios for VSGA and neonatal death in the post-ART expansion period were generated using logistic regression models

§Abbreviations: PTD, preterm delivery (<37 weeks gestational age); VPTD, very preterm delivery (<32 weeks gestational age); SGA, small for gestational age (<10^th^ percentile for gestational age); VSGA, very small for gestational age (<3^rd^ percentile for gestational age).

**Figure S1. Proportions of Adverse Birth Outcomes Among Non-Citizens Without HIV Pre- vs. Post-ART Policy Expansion**

Abbreviations: PTD, preterm delivery (<37 weeks gestational age); VPTD, very preterm delivery (<32 weeks gestational age); SGA, small for gestational age (<10^th^ percentile for gestational age); VSGA, very small for gestational age (<3^rd^ percentile for gestational age). This figure displays the changes in the adverse birth outcomes among non-citizens after the ART policy expansion.

**Table S3. Adverse birth outcomes in Botswana among citizens and non-citizens, before and after ART expansion in December 2019 among pregnant people with HIV [singleton births and primiparous]**

|  | August 2014 - November 2019 | | | | December 2019 – September 2021 | | | |
| --- | --- | --- | --- | --- | --- | --- | --- | --- |
|  | Citizen  n =5,547 (%) | Non-Citizen  n = 118 (%) | RR,  95% CI | aRR^†^,  95% CI | Citizen  n = 1,949 (%) | Non-Citizen  n= 72 (%) | RR,  95% CI | aRR^†^,  95% CI |
| PTD^‡^ | 984 (17.7) | 35 (29.7) | 1.71 (1.26, 2.21) | 1.76 (1.08, 2.82) | 294 (15.1) | 14 (19.4) | 1.34 (0.78, 2.06) | 1.33 (0.67, 2.47) |
| VPTD | 214 (3.9) | 14 (11.9) | 3.14 (1.80, 5.00) | 2.97 (1.42, 5.69) | 69 (3.5) | 2 (4.2) | 0.82 (0.14, 2.51) | 0.68 (0.11, 2.42) |
| SGA | 1,322 (23.8) | 23 (19.5) | 0.85 (0.57, 1.19) | 0.94 (0.56, 1.54) | 391 (20.1) | 11 (15.3) | 0.78 (0.42, 1.28) | 0.72 (0.35, 1.39) |
| VSGA | 542 (9.8) | 9 (7.6) | 0.81 (0.39, 1.42) | 0.81 (0.35, 1.61) | 162 (8.3) | 3 (4.2) | 0.52 (0.13, 1.31) | 0.61 (0.14, 1.72) |
| Stillbirth | 133 (2.4) | 5 (4.2) | 1.77 (0.63, 3.79) | 2.39 (0.80, 5.74) | 57 (2.9) | 0 (0.0) | N/A | N/A |
| Neonatal Death | 72 (0.02) | 8 (6.8) | 5.37 (2.43, 10.17) | 4.34 (1.61, 10.25) | 21 (1.1) | 0 (0.0) | N/A | N/A |

†Adjusted for maternal age, education, occupation, and marital status.

‡ Abbreviations: PTD, preterm delivery (<37 weeks gestational age); VPTD, very preterm delivery (<32 weeks gestational age); SGA, small for gestational age (<10^th^ percentile for gestational age); VSGA, very small for gestational age (<3^rd^ percentile for gestational age).

Note: Due to convergence issues, logistic regression models were generated for VSGA in post-ART period.

Table S4. Log-Binomial Regression models for **pregnant persons with HIV** when assigning missing values to lowest variable value [modified table]

|  | | August 2014 - November 2019 | | | | December 2019 – September 2021 | | | |
| --- | --- | --- | --- | --- | --- | --- | --- | --- | --- |
|  | | RR,  95% CI | | aRR^†^,  95% CI | | RR,  95% CI | | aRR^†^,  95% CI | |
| PTD^‡^ | | 1.22 (1.08, 1.37) | | 1.29 (1.14, 1.45) | | 0.87 (0.70, 1.07) | | 0.88 (0.70, 1.08) | |
| VPTD | | 1.72 (1.35, 2.15) | | 1.84 (1.43, 2.32) | | 0.89 (0.53, 1.38) | | 0.78 (0.46, 1.23) | |
| SGA | | 0.74 (0.63, 0.87) | | 0.73 (0.62, 0.86) | | 0.86 (0.69, 1.06) | | 0.86 (0.68, 1.06) | |
| VSGA | | 0.77 (0.58, 0.99) | | 0.74 (0.56, 0.95) | | 0.86 (0.59, 1.21) | | 0.81 (0.55, 1.15) | |
| Stillbirth | | 1.46 (1.05, 1.97) | | 1.34 (0.95, 1.82) | | 0.95 (0.54, 1.52) | | 0.84 (0.48, 1.37) | |
| Neonatal Death | | 1.85 (1.22, 2.67) | | 1.72 (1.12, 2.52) | | 1.43 (0.71, 2.40) | | 1.30 (0.63, 2.37) | |

†Adjusted for maternal age, education, occupation, and marital status.

‡ Abbreviations: PTD, preterm delivery (<37 weeks gestational age); VPTD, very preterm delivery (<32 weeks gestational age); SGA, small for gestational age (<10^th^ percentile for gestational age); VSGA, very small for gestational age (<3^rd^ percentile for gestational age).

Note: Due to convergence issues, logistic regression models were generated for aRRs.

Table S5. Log-Binomial Regression models for **pregnant persons without HIV** when assigning missing values to lowest variable value [modified table]

|  | | August 2014 - November 2019 | | | | December 2019 – September 2021 | | | |
| --- | --- | --- | --- | --- | --- | --- | --- | --- | --- |
|  | | RR,  95% CI | | aRR^†^,  95% CI | | RR,  95% CI | | aRR^†^,  95% CI | |
| PTD^‡^ | | 1.08 (0.99, 1.16) | | 1.15 (1.05, 1.25) | | 1.08 (0.97, 1.19) | | 1.08 (0.97, 1.20) | |
| VPTD | | 1.13 (0.94, 1.34) | | 1.19 (0.99, 1.43) | | 1.24 (0.97, 1.56) | | 1.24 (0.95, 1.58) | |
| SGA | | 0.77 (0.70, 0.85) | | 0.83 (0.75, 0.91) | | 0.81 (0.72, 0.91) | | 0.84 (0.74, 0.95) | |
| VSGA | | 0.82 (0.69, 0.96) | | 0.85 (0.72, 1.00) | | 0.83 (0.67, 1.01) | | 0.84 (0.67, 1.03) | |
| Stillbirth | | 1.28 (1.03, 1.56) | | 1.15 (0.92, 1.42) | | 1.33 (1.03, 1.68) | | 1.19 (0.91, 1.54) | |
| Neonatal Death | | 1.17 (0.87, 1.54) | | 1.20 (0.88, 1.60) | | 1.38 (0.95, 1.92) | | 1.36 (0.92, 1.95) | |

†Adjusted for maternal age, education, occupation, and marital status.

‡ Abbreviations: PTD, preterm delivery (<37 weeks gestational age); VPTD, very preterm delivery (<32 weeks gestational age); SGA, small for gestational age (<10^th^ percentile for gestational age); VSGA, very small for gestational age (<3^rd^ percentile for gestational age).

Note: Due to convergence issues, logistic regression models were generated for aRRs.

Table S6. Proportions of At Least One ANC Visit During Pregnancy Among Non-Citizens by HIV Status

|  | **Without HIV** | |  | **With HIV** | |  |
| --- | --- | --- | --- | --- | --- | --- |
|  | **Pre-ART Expansion**  **(n = 3,463) (%)** | **Post-ART Expansion**  **(n = 2,296) (%)** | **P-Value** | **Pre-ART Expansion**  **(n = 954) (%)** | **Post-ART Expansion**  **(n = 562) (%)** | **P-value** |
| **At least 1 ANC Visit** |  |  | 0.23 |  |  | <0.001 |
| No | 386 (11.1) | 233 (10.1) |  | 180 (18.9) | 63 (11.2) |  |
| Yes | 3038 (87.7) | 2037 (88.7) |  | 756 (79.2) | 490 (87.2) |  |
| Missing | 39 (1.1) | 26 (1.1) |  | 18 (1.9) | 9 (1.6) |  |
